# Effect of periodic nursing interventions on improving compliance behavior and health status in long-term Hemodialysis patients

**DOI:** 10.12669/pjms.40.9.9076

**Published:** 2024-10

**Authors:** Yan Wang, Jiakang Sun, Fei Guo, Deheng Wan

**Affiliations:** 1 Yan Wang, Department of Nephrology, Qinhuangdao Haigang Hospital, Qinhuangdao 066000, Hebei, China; 2 Jiakang Sun, Department of Nephrology, Qinhuangdao Haigang Hospital, Qinhuangdao 066000, Hebei, China; 3 Fei Guo, Department of Nephrology, Qinhuangdao Haigang Hospital, Qinhuangdao 066000, Hebei, China; 4 Deheng Wan, Department of Nephrology, Qinhuangdao Haigang Hospital, Qinhuangdao 066000, Hebei, China

**Keywords:** Periodic Nursing, Hemodialysis Patients, Compliance Behavior, Health Status

## Abstract

**Objective:**

To investigate the effect of periodic nursing interventions on improving compliance behavior and health status in long-term hemodialysis patients.

**Method:**

This was retrospective study. A total of 88 patients on long-term hemodialysis admitted to Qinhuangdao Haigang Hospital from January 2022 to April 2023 were randomly divided into the control group and the intervention group, who received routine hemodialysis nursing and periodic nursing interventions, respectively. Afterward, compliance behavior and health status were compared between the two groups, along with a comprehensive assessment of the health status using renal function indexes, complication rates, and anxiety and depression scores.

**Result:**

Before nursing, no significant difference was observed in each index between the two groups (p > 0.05). After 16 months of nursing, the score of compliance behavior in the intervention group (88.68 ± 6.27) was significantly higher than that in the control group (70.91 ± 7.48) (p< 0.05), with the renal function indexes of BUN and SCr lower than those in the control group (p< 0.05). Meanwhile, the intervention group saw higher SAS and SDS scores than the control group (p< 0.05).

**Conclusion:**

Periodic nursing interventions may effectively improve the compliance behavior and health status of patients, enhance the efficacy of hemodialysis treatment, and improve the quality of life of patients.

## INTRODUCTION

As one of the major threats to the health of the global population, Chronic kidney disease (CKD) affects 11%~13% of people worldwide,^1^ with 568 of every million people in China suffering from end-stage renal disease(ESRD), which seriously affects the normal life of patients.^2^ Hemodialysis is the major treatment option for ESRD^3^ and helps to delay the progression of the disease and prolong the patient’s life, thus significantly boosting the survival rates.^4^ Due to the long cycle of hemodialysis treatment, it easily leads to complications such as acid-base and electrolyte balance disorders^5^, hypotension,^6^ muscle weakness,^7^ cardiovascular disease,^8^,^9^ malnutrition,^10^ anxiety, and depression.^11^ Specifically, during long-term hemodialysis treatment, the incidence of hypotension and malnutrition is 15%~50%^12^ and 40%~70%, respectively,^13^ with more than 60% of patients failing to comply with diet and fluid intake restrictions,^14^ which brings numerous adverse physical and psychological effects to patients.

Patients with ESRD are frail and need to endure the long-term pain from treatment and a variety of complications, as well as the pressure of hefty medical costs, resulting in many patients being unable to effectively cooperate or even giving up treatment.^15^ Since renal diseases result from multifaceted factors, the dialysis treatment process also requires the cooperation of multi-field teams. To improve the effect of hemodialysis treatment, clinicians, nursing staff, psychologists, and dietitians contributed joint efforts in developing a scientific nursing intervention regimen to implement support or intervention according to the specific needs of patients at different stages, so as to improve the compliance behavior and treatment efficacy of patients.^16^ Therefore, this study focused on periodic nursing intervention for long-term hemodialysis patients and to investigate the effect of periodic nursing interventions on improving compliance behavior and health status in long-term hemodialysis patients.

## METHODS

This was a retrospective study. Eighty eight long term hemodialysis patients admitted to Qinhuangdao Haigang Hospital from January 2022 to April 2023 were selected and divided into the control group (n=44) and the intervention group (n=44) according to different nursing methods.

### Ethical Approval:

The study was approved by the Institutional Ethics Committee of Qinhuangdao Haigang Hospital (No.:20211119; date: November 19, 2021), and written informed consent was obtained from all participants.

### Inclusion criteria:

- Patients clinically diagnosed with ESRD.
- Patients who had received hemodialysis treatment for over three months, 2-3 times a week, and took part in the study on periodic nursing interventions for the first time.
- Patients and their family members voluntarily signed the relevant written documents.
- Patients with normal communication comprehension ability to ensure the accuracy and reliability of research data.

### Exclusion criteria:

- Patients with mental illness or normal communication ability.
- Patients with malignant tumors, organ dysfunction, bleeding disorders, etc.
- Patients who had undergone kidney transplantation.
- Patients who couldn’t take care of themselves.
- Patients with severe acute and chronic infectious diseases.

The control group included 22 males and 22 females aged 40 ~ 71, with an average age of (57.39 ± 7.61), while the intervention group included 22 males and 22 females aged 43~70, with an average age of (55.02 ± 7.49). No significant difference was observed in the general data of long-term hemodialysis patients between the two groups (p > 0.05).

The Control group: Routine nursing measures were implemented, i.e., preoperative explanation of the disease, postoperative nursing, daily diet and medication instructions, routine psychological counseling, etc., as well as recording changes in various vital signs of patients and creating health records.

A periodic nursing intervention team was formed, which consisted of clinicians, nurses, nutrition experts, and psychologists. The treatment course of patients was divided into no-intention stage, intention stage, preparation stage, action stage, and maintenance stage. Meanwhile, the intervention team regularly conducted one-on-one data testing and analysis for each patient, classified the patient’s current treatment stage, established health indicator records, and developed or adjusted the patient’s intervention nursing regimen. Patients were assessed once a month for health indicators, with secondary adjustments made to the intervention nursing regimen for patients who did not meet the criteria.

### No-intention stage and intention stage:

Collective lectures were conducted (twice a week, 30 Minutes each time), with one-on-one counseling provided (once a week, 30 minutes each time). The pre-intervention compliance behavior was scored, with renal function indexes tested and depression scored to establish an intervention nursing record. Additionally, patient chat groups were established to improve the efficiency of Q&A for patients.

### Preparation stage and action stage:

Lectures on daily care, proper medication use, healthy diet and exercise, weight control, and the importance and methods of appropriate water intake were regularly conducted (twice a week, 30 Minutes each time). Follow-up via phone or home visits (once a week) were conducted while logging patient data and answering patient’s questions (30 Minutes/person), so as to timely identify changes in the patient’s condition, provide phone-based guidance or face-to-face consultations, and adjust individual intervention regimens.

### Maintenance stage:

Patients were scored for compliance behavior, tested for renal function indexes, and scored for depression. Targeted guidance was provided for patients with complications and maintenance difficulties. Moreover, patient communication activities were organized (once a week, 60 Minutes/time) and one-on-one counseling was provided to reduce the negative emotions of patients, along with regular health check-ups.

### Outcome Indexes:

- Compliance behavior. Combined with relevant data, the team self-designed a questionnaire on the compliance behavior of patients on maintenance hemodialysis, covering a total of 6 dimensions, i.e., fluid intake compliance, regular clinic visits, proper medication use, balanced diet, regular exercise, and weight control. Each dimension contains five questions, totaling 30 questions, and each question was scored from 1 to 4, with a total score of 30 to 120. The higher the score, the better the patient’s compliance behavior.
- Renal function indexes. BUN and SCr were measured by the urease method and picric acid method, respectively.
- Incidence of complications. The incidence of hypotension, vascular access infection, hyperkalemia, hyperphosphatemia, malnutrition, fistula occlusion, skin itching, and cardiac lesions were counted in both groups, respectively.
- Patient anxiety and depression. Self-Rating Anxiety Scale (SAS) and Self-Rating Depression Scale (SDS) were used for assessment, respectively. SAS score<50 was considered normal, and SDS score<53 indicated no anxiety. The higher the score, the more severe the anxiety and depression.

### Statistical Methods:

Data analysis was conducted using SPSS 22.0 software. The count data was expressed as percentages (%), and the χ^2^ test was used for comparison. The measurement data were expressed as *χ̅*±*S*. P< 0.05 was considered statistically significant.

## RESULTS

As shown in Table-I, after regular periodic nursing interventions, fluid intake compliance, regular clinic visits, proper medication use, balanced diet, regular exercise, weight control, and the overall scores of patients in the intervention group were higher than those in the control group, and the differences were statistically significant (p < 0.05). Therefore, the periodic nursing interventions effectively improved the compliance behavior of long-term hemodialysis patients.

**Table-I:** Comparison of Compliance Behavior Scores (N=44,`χ+s).

Compliance Behavior	Control Group		Intervention Group	

	Pre-intervention	Post-intervention	Pre-intervention	Post-intervention
Fluid intake compliance	9.02±3.17	7.89±2.61	12.86±2.79	16.71±2.47^a^
Regular clinic visits	7.52±2.41	7.91±2.72	11.45±2.57	13.48±2.88^a^
Proper medication use	12.07±2.21	12.48±1.98	13.82±2.12	16.91±2.13^a^
Balanced diet	8.39±2.09	8.11±2.01	10.91±2.40	15.11±2.26^a^
Regular exercise	9.68±3.39	10.55±3.62	11.64±3.21	13.86±3.05^a^
Weight control	11.09±2.92	11.73±2.97	11.59±2.42	14.11±3.65^a^
Overall	59.48±7.52	57.89±5.50	70.91±7.48	88.68±6.27^a^

***Note:*** Compared with the control group after the intervention,

a p< 0.05.

In Table-II the BUN and SCr values in the intervention group were lower than those in the control group after nursing interventions, and the difference was statistically significant (p < 0.05). As shown in Table-III, the intervention group saw a lower incidence of each complication than the control group after nursing interventions, and the difference had statistical significance (p < 0.05).

**Table-II:** Comparison of Renal Function Indexes (N=44,`χ+s).

Group	BUN (mmol/L)		SCr (μmol/L)	

	Pre-intervention	Post-intervention	Pre-intervention	Post-intervention
Control	24.57±2.35	17.21±1.28	490.41±10.77	311.39±10.81
Intervention	24.86±2.51	13.51±0.90^b^	492.32±9.90	274.80±14.01^b^

***Note:*** Compared with the control group after the intervention,

b p< 0.05.

**Table-III:** Comparison of Complication Rates [n (%), N=44].

Complication	Control Group	Intervention Group
Hypotension	2	1
Vascular access infection	2	2
Hyperkalemia	1	0
Hyperphosphataemia	2	0
Malnutrition	2	0
Fistula occlusion	1	0
Skin pruritus	2	2
Cardiac lesion	1	0
Overall complication rate	13(29.54)	5(11.36)

***Note:*** Comparison of incidence of long-term hemodialysis complications between the control and intervention groups, χ2 = 4.470, p< 0.05.

No significant difference was observed in SAS and SDS scores before intervention between the two groups (p > 0.05); after intervention, SAS and SDS scores decreased in both groups, with the intervention group seeing significantly lower scores than the control group, and the difference was statistically significant (p < 0.05). (Table-IV).

**Table-IV:** Comparison of Anxiety & Depression Scores (N=44,`χ±s).

Group	SAS Score		SDS Score	

	Pre-intervention	Post-intervention	Pre-intervention	Post-intervention
Control	60.11±3.10	47.41±2.95	65.86±4.85	51.02±4.81
Intervention	60.23±2.68	34.91±4.17^c^	65.16±6.39	46.57±3.29^c^

***Note:*** Compared with the control group after intervention,

c p< 0.05.

## DISCUSSION

The findings of this study showed that before nursing, no significant difference was observed in each index between the two groups (p> 0.05). After 16 months of periodic nursing interventions, the scores of compliance behavior in the intervention group were significantly higher than those in the control group. Meanwhile, the intervention group saw lower BUN and SCr values of renal function indexes and higher SAS and SDS scores of the emotional indexes than the control group, and the differences were statistically significant (p< 0.05), indicating excellent results.

Statistics show that there have been more than 100 million patients with chronic kidney disease(CKD) in China, making it one of the serious diseases that threaten the health of people across the country.^17^ Chronic kidney disease(CKD) gradually develops into ESRD, leading to problems such as cardiopulmonary failure, electrolyte imbalance, malnutrition, weight gain, and vascular calcification.^18^ Currently, hemodialysis is a critical treatment for ESRD, in which blood purification can help patients remove metabolic wastes, toxins, and excessive fluids from the body. However, long-term hemodialysis treatment easily results in the loss of some beneficial proteins and cells, inducing complications such as malnutrition and hypotension.^19^,^20^

Meanwhile, the patient’s factors also have an important impact on the effect of long-term hemodialysis treatment. Factors such as excessive fluid intake, a diet high in oil and salt, improper medication use, withdrawal from or even discontinuation of treatment, and lack of exercise all adversely affect the quality of hemodialysis and easily lead to an increased incidence of various complications. Moreover, many patients find it difficult to cope with the physical, economic, and psychological pressure, resulting in a gradual decline in compliance behavior and even giving up treatment. Therefore, effective intervention and nursing measures from the medical and nursing teams play an important role in controlling the condition of long-term hemodialysis patients and improving their prognosis.

Currently, routine nursing mainly focuses on education and medication guidance, lacking targeted and scientific intervention for patients at different stages. This study investigated the effect of periodic nursing interventions on compliance behavior and health status of long-term hemodialysis patients. At the early stage of hemodialysis treatment, patients experienced symptom relief and improved health conditions, resulting in higher compliance with routine medical and nursing education and guidance. However, as the hemodialysis treatment progressed, patients developed a sense of psychological inertia over the long-term process, leading to a decline in compliance behavior and a weakened physical condition.

Additionally, with the increasing economic and psychological burden from the long-term treatment, the more proMinutesently negative emotions such as sensitivity, irritability, anxiety, and depression, and more stressed family and social relationships, the initial guidance program was unable to meet the treatment needs of patients. Therefore, it is necessary to regularly conduct face-to-face consultations, follow-up via phone or home visits, and implement measures such as conducting surveys on compliance behavior, health indicator assessments, and psychological evaluations to promptly identify changes in patient’s condition.

Meanwhile, for patients with poor treatment outcomes, intervention regimens, and nursing approaches should be adjusted, along with the disseMinutesation of knowledge on long-term hemodialysis treatment and nursing, strengthening of nutritional support, enhancement of patient physical condition, emphasis on the importance of fluid intake and weight control through targeted counseling, creation of patient chat groups to facilitate peer communication, and organizing of offline activities for patients to increase their social engagement and improve their negative emotions. These interventions are designed to improve the compliance behavior, health status, and quality of life of patients.

Afterward, once moving on to the maintenance stage of hemodialysis treatment, most patients show satisfactory treatment outcomes. However, for a small number of patients with complications, it is necessary to collect and analyze treatment data, assist in identifying the causes, increase the frequency of interventions, and further adjust the intervention regimen.

### Limitations:

In this study, the compliance behavior and health status of hemodialysis patients were investigated for only 16 months. Generally, patients with ESRD need to undergo hemodialysis for many years. Therefore, further follow-up and discussion are needed to explore the effect of periodic nursing interventions. Meanwhile, due to the fact that periodic nursing interventions require more healthcare workers and longer periods of treatment than usual nursing measures, further research is also needed to reduce the medical costs for patients.

## CONCLUSION

Periodic nursing interventions for long-term hemodialysis patients may be beneficial for improving the self-care ability of patients and may lead to a positive impact on improving the treatment efficacy and the quality of life of patients.

### Authors’ Contributions:

**YW:** Carried out the studies, data collection, a drafted the manuscript, are responsible and accountable for the accuracy or integrity of the work.

**JS FG DW:** Performed the statistical analysis and participated in its design.

All authors read and approved the final manuscript.
